# Magnetic Resonance and Vibrational Spectroscopy and Imaging in Food Analysis

**DOI:** 10.3390/molecules27248831

**Published:** 2022-12-13

**Authors:** Luiz Alberto Colnago, Luis E. Rodriquez-Saona, Zeev Wiesman

**Affiliations:** 1 Brazilian Corporation for Agricultural Research, Embrapa Instrumentation, P.O. Box 741, São Carlos 13560-970, SP, Brazil; 2 Department of Food Science and Technology, The Ohio State University, Columbus, OH 43210, USA; 3 Department of Biotechnology Engineering, Ben Gurion University of the Negev, Beer Sheva 84105, Israel

In the past two decades, there have been remarkable changes in the way we analyze the physical, chemical, and sensory properties of fresh and processed food products, with the progressive replacement of traditional wet analytical methods (destructive, laborious, time-consuming, and requiring the use of hazardous chemicals) with new, fast, non-destructive physical methods where the analysis is performed in a single step, after validation, and without the use of chemical reagents. With new magnetic resonance and vibrational spectroscopic and imaging methods, food analyses have become faster, more accurate, and better able to determine several parameters per analysis without destroying the sample (non-destructive methods), or in some cases without even having to open their sealed packages (non-invasive methods). Many advances also include the development of new equipment (some even portable), easier access, greater sensitivity, better resolution, lower detection and quantification limits, and the use of computational procedures known as chemometrics or machine learning methods. Currently, most of the food analyses with these technologies have been performed in laboratories, but analyses in fields, factories and warehouses are gaining momentum, and analyses by consumers in supermarkets are just around the corner.

This Special Issue, “*Magnetic Resonance and Vibrational Spectroscopy and Imaging in Food Analysis*”, published in the journal *Molecules*, includes ten original papers: seven papers using nuclear magnetic resonance spectroscopy and relaxometry (NMR) and imaging (MRI), and three papers using near- and middle-infrared spectroscopy and hyperspectral imaging. The NMR papers are as follows: Sørensen et al. [1] demonstrate the use of portable and easy-to-use NMR instruments to measure fat and protein content in milk, on site. Salvador et al. [2] show the use of high- and low-field NMR spectroscopy to monitor the effect of UV-C light on the bean-darkening process. Machado et al. [3] demonstrate the potential of low-field NMR relaxometry to predict fat, moisture and solid fat content in soft cheese in commercial packages. Oshester et al. [4] use a low-field NMR spectrometer to monitor the oxidation status of edible oil via the measurement of the oil self-diffusion coefficient. Uguz et al. [5] show the potential of solid-state and spin diffusion pulse sequences in food quality control using a time domain NMR spectrometer. The MRI papers are as follows: Serial et al. [6] study the time-dependent flow behavior of concentrated egg yolk emulsions via MRI and unravel the effects caused by viscous friction during shear. Kerr et al. [7] demonstrate via MRI that high pressure reduces the infusion time 100 times when compared with infusion at an ambient pressure.

The infrared spectroscopy and imaging papers are as follows: Aykas et al. [8] develop a rapid, accurate method to detect acrylamide content in par-frozen French fries using a portable Infrared spectrometer. Ren et al. [9] use Fourier transform infrared spectroscopy to study the stability and interactions of anthocyanins and whey proteins. Finally, Jiang et al. [10] develop a hyperspectral method, from 400 to 1000 nm, to rapidly and accurately discriminate the maturity stages of *Camellia oleifera* fruits.

We hope that this Special Issue provides useful information about the potential of nuclear magnetic resonance and infrared spectroscopies and imaging as simple, rapid and non-destructive procedures to monitor several physical and chemical parameters that are important to understand food properties and food quality.
